# Predicting tuberculosis drug efficacy in preclinical and clinical models from *in vitro* data

**DOI:** 10.1016/j.isci.2025.111932

**Published:** 2025-01-30

**Authors:** Janice J.N. Goh, Anu Patel, Bernard Ngara, Rob C. van Wijk, Natasha Strydom, Qianwen Wang, Nhi Van, Tracy M. Washington, Eric L. Nuermberger, Bree B. Aldridge, Christine Roubert, Jansy Sarathy, Véronique Dartois, Rada M. Savic

**Affiliations:** 1 Department of Bioengineering and Therapeutic Sciences, University of California, San Francisco, San Francisco, CA, USA; 2 Department of Molecular Biology and Microbiology, Tufts University School of Medicine, and Stuart B. Levy Center for Integrated Management of Antimicrobial Resistance Boston, Boston, MA, USA; 3 Department of Biomedical Engineering, Tufts University School of Engineering, Medford, MA, USA; 4 Center for Tuberculosis Research, Department of Medicine, Johns Hopkins University School of Medicine, Baltimore, MD, USA; 5 Evotec ID (LYON) SAS, Lyon, France; 6 Sanofi R&D, Infectious Diseases TSU, 31036 Toulouse, France; 7 Center for Discovery and Innovation, Hackensack Meridian School of Medicine, Hackensack Meridian Health, Nutley, NJ, USA

**Keywords:** bioinformatics, biological sciences, natural sciences, pharmacoinformatics, pharmacology

## Abstract

Multiple *in vitro* potency assays are used to evaluate compounds against *Mycobacterium tuberculosis*, but a consensus on clinically relevant assays is lacking. We aimed to identify an *in vitro* assay signature that predicts preclinical efficacy and early clinical outcome. Thirty-one unique *in vitro* assays were compiled for 10 TB drugs. *In vitro* EC_50_ values were compared to pharmacokinetic-pharmacodynamic (PK-PD)-model-derived EC_50_ values from mice evaluated via multinomial regression. External validation of best-performing *in vitro* assay combinations was performed using five new TB drugs. Best-performing assay signatures for acute and subacute infections were described by assays that reproduce conditions found in macrophages and foamy macrophages and chronic infection by the *ex vivo* caseum assay. Subsequent simulated mouse bacterial burden over time using predicted *in vivo* EC_50_ was within 2-fold of observations. This study helps us identify clinically relevant assays and prioritize successful drug candidates, saving resources and accelerating clinical success.

## Highlights

• In TB, it is unknown which potency assays prioritize *in vivo* compound efficacy
• We made a PK-PD platform using *in vivo* potency data to identify clinically relevant assays
• This platform could also predict monotherapy outcomes in preclinical and clinical studies
• This helps prioritize promising drug candidates and save resources in drug development

## Introduction

Tuberculosis (TB) was the top killer among infectious diseases globally as of 2022.^1^ A standard therapy consisting of a cocktail of four drugs exists,^2^ but long treatment duration and strict adherence requirements make cure difficult to achieve.^3^ Thus, there is an urgent need for the development of new drugs that can shorten treatment duration. Many novel drug candidates have emerged in recent years^4^; however, the clinical trial process is often long and expensive. Therefore, it is essential to better prioritize which drugs are the most likely to succeed clinically.

*In vitro* assays are typically used to test a new compound for activity prior to animal studies. Traditionally, the minimum inhibitory concentration (MIC) is used to determine a drug’s potency and efficacy against *Mycobacterium tuberculosis* (Mtb).^5^ However, MIC assays are often carried out in a nutrient-rich media that promotes rapid bacterial growth and are not representative of the physiological conditions in which Mtb grows in human hosts. Furthermore, throughout treatment, Mtb growth slows, making it less susceptible to drug treatment.^6^^,^^7^ Therefore, novel *in vitro* assays have since been developed to better mimic this slow-growing, “persister” state, such as depriving Mtb of key nutrients or oxygen or adding immune cells to simulate what happens in an actual infection.^8^ There is not yet consensus on which of these assays are most predictive of *in vivo* drug efficacy, and thus most informative, for prioritizing preclinical and clinical candidates.

Previously, we developed an integrated pharmacokinetic-pharmacodynamic (PK-PD) model describing bacterial dynamics that allows us to estimate the *in vivo* potency (EC_50_) of a drug in mice after accounting for the adaptive immune effect.^9^ EC_50_ is defined as the drug concentration needed to achieve 50% of maximal response. In an *in vivo* model, drug concentration is dynamic and changes over time. However, by accounting for this using PK-PD modeling, we can integrate both drug concentration and pharmacodynamic response over time into a single model and estimate an *in vivo* EC_50_, similar to *in vitro* EC_50_, as a single concentration. We found that EC_50_ from mice was portable to humans and could be used to estimate clinical early bactericidal activity (EBA) outcomes in Phase 2a trials. Using *in vivo* EC_50_ from mice as a standard, we aimed to ask two questions: (1) Which *in vitro* assays, or combination of assays, are the most useful for predicting *in vivo* EC_50_? and (2) Are these *in vitro* correlates of *in vivo* EC_50_ useful for the prediction of both *in vivo* preclinical efficacy and clinical efficacy? Knowing which *in vitro* assays are most informative for the prediction of outcomes in preclinical *in vivo* models and clinical outcomes will help us to streamline compound progression and accelerate drug development.

## Results

### Compilation of a rich *in vitro* assay dataset

**Figure 1:**
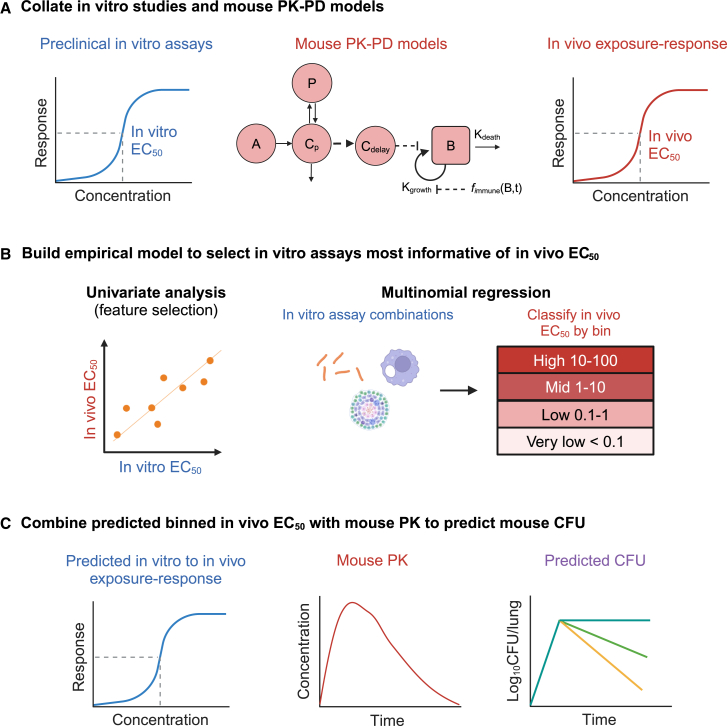
A three-step pipeline to translate *in vitro* potency (EC_50_) to *in vivo* mouse EC_50_ and *in vivo* efficacy when combined with a pharmacokinetic-pharmacodynamic (PK-PD) model (A) *In vitro* assays were collected from literature and from collaborators for 10 drugs of interest: bedaquiline (BDQ), delamanid (DLM), ethambutol (EMB), isoniazid (INH), linezolid (LZD), moxifloxacin (MXF), pretomanid (PMD), pyrazinamide (PZA), rifampicin (RIF), and rifapentine (RPT). Mouse PK-PD models with a baseline describing bacterial dynamics were also previously built for these drugs. (B) Univariate linear regression was first carried out to understand the individual relationships between *in vitro* EC_50_ and *in vivo* EC_50_ derived from mouse PK-PD models. A multinomial regression was then built to find the least number of *in vitro* assays with the best accuracy for predicting *in vivo* EC_50_. (C) Predicted *in vivo* EC_50_ was used to make a new exposure-response relationship in the mouse PK-PD model to predict the bacterial colony-forming units (CFUs) over time profile in mouse.

**Figure 2:**
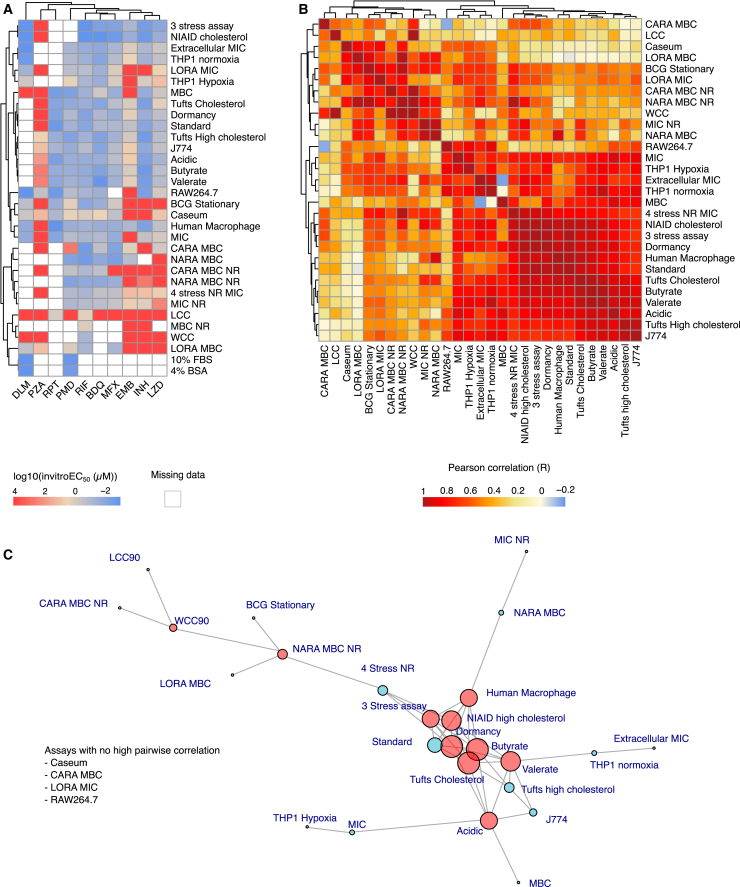
*In vitro* assay description and correlation (A) Overview of all 31 *in vitro* assays across 10 drugs of interest, clustered by their *in vitro* potency (EC_50_). Missing data were denoted as an empty white square, whereas inactive drugs in an assay were arbitrarily assigned a value of 9999. (B) Pairwise correlations between *in vitro* assays clustered by their Pearson correlation value (R) show many high positive correlations between *in vitro* assays. (C) Network plot of high pairwise correlations between *in vitro* assays. *In vitro* assays with absolute pairwise correlations higher than 0.9 were joined by an edge in the network plot. Assays with six or more edges, or with the greatest number of edges in their cluster, filled in red, were chosen as representative of the many highly correlated assays. Table 2 also lists every significant pairwise correlation among the assays with high correlation. Assays that had no high pairwise correlations were also selected as features for further analysis. BDQ, bedaquiline, DLM, delamanid, EMB, ethambutol, INH, isoniazid, LZD, linezolid, MXF, moxifloxacin, PMD, pretomanid, PZA, pyrazinamide, RIF, rifampicin, and RPT, rifapentine.

We compiled a rich dataset of 31 unique assays tested on 10 key first- and second-line drugs with corresponding PK-PD models and clinical data available (Figures 1 and 2A). These drugs were bedaquiline (BDQ), delamanid (DLM), ethambutol (EMB), isoniazid (INH), moxifloxacin (MFX), linezolid (LZD), pretomanid (PMD), pyrazinamide (PZA), rifampicin (RIF), and rifapentine (RPT). Of these 10 drugs, 7 had efficacy data in mouse acute models (EMB, INH, LZD, MXF, PMD, PZA, and RIF), 8 in subacute models (BDQ, DLM, INH, LZD, MXF, PMD, PZA, and RIF), and 7 in chronic models (INH, LZD, MXF, PMD, PZA, RIF, and RPT). Most of the assays only reported drug potencies rather than a full dose-response curve. Hence, we focused on EC_50_ rather than E_max_ to generate the exposure response.

Our assumption of a Hill coefficient of 1, which allowed us to easily convert all potencies to EC_50_, held true in our analysis. When comparing models with and without a Hill coefficient using a nested F test, the reported nested F value of 0.600 showed statistical insignificance.

To categorize the assays as a function of their biological relevance at the various sites of disease, we created four broad categories: (1) nutrient-rich media conditions representing extracellular rapidly replicating Mtb, (2) essential nutrient substitution or deletion slowing Mtb replication, (3) macrophage assays reflecting the involvement of the immune system slowing down Mtb growth to a more persistent state during acute infection, and (4) *ex vivo* assays representing the necrotic lesion core formed in severe cases of TB infection. Out of these 31 assays, 7 were under nutrient-rich conditions (e.g., MIC in Middlebrook 7H9 medium), 18 were a change in culture condition, via either a media formulation or oxygen deprivation, 5 were macrophage assays involving the uptake of Mtb into an immune cell, and 1 was an *ex vivo* assay using harvested caseum from New Zealand White rabbits (Figure 2A). Three assays—minimum bactericidal activity under non-replicating conditions (MBC NR) and two assays designed to quantify the impact of nonspecific drug binding to serum (rich medium plus 10% fetal bovine serum [FBS] or 4% bovine serum albumin [BSA])—did not have assay information for at least three drugs of interest and were discarded (Figure 2A). The full list of assays and data sources is displayed in Table 1.

**Table 1:** *In vitro* assays compiled and their sources

Assay type	Source	References	Assay classification/bacterial population
10% FBS MIC	TBDA	Upton et al. 2014^10^	Nutrient-rich, nonspecific drug binding/fast-growing
3 Stress Assay	TBDA	Early et al. 2019^11^	Nutrient substitution/slow-growing
4% BSA MIC	TBDA	Upton et al. 2014^10^	Nutrient-rich, nonspecific drug binding/fast-growing
4 Stress NR CARA	TBDA	Gold et al. 2015^12^	Nutrient substitution/slow-growing
4 Stress NR MIC	TBDA	Gold et al. 2015^12^	Nutrient substitution/slow-growing
BCG Stationary	TBDA	Quezada 2019^13^	Nutrient substitution/slow-growing
CARA MBC	TBDA	Gold et al. 2015^12^	Nutrient-rich/fast-growing
Caseum	Dartois lab, HMH	Sarathy et al. 2018^14^	*Ex vivo* caseum/slow-growing
Human Macrophage	Sarathy lab, HMH	Lanni et al. 2023^15^	Macrophage/slow-growing
Acidic	Aldridge lab, TU	Larkins-Ford et al. 2021^16^	Nutrient substitution/slow-growing
Butyrate	Aldridge lab, TU	Larkins-Ford et al. 2021^16^	Nutrient substitution/slow-growing
Tufts Cholesterol	Aldridge lab, TU	Larkins-Ford et al. 2021^16^	Nutrient substitution/slow-growing
Tufts High Cholesterol	Aldridge lab, TU	Larkins-Ford et al. 2021^16^	Nutrient substitution/slow-growing
Dormancy	Aldridge lab, TU	Larkins-Ford et al. 2021^16^	Nutrient substitution/slow-growing
J774	Aldridge lab, TU	Larkins-Ford et al. 2021^16^	Macrophage/slow-growing
Tufts Standard	Aldridge lab, TU	Larkins-Ford et al. 2021^16^	Nutrient-rich/fast-growing
Valerate	Aldridge lab, TU	Larkins-Ford et al. 2021^16^	Nutrient substitution/slow-growing
THP-1 Hypoxia	Evotec	S. Souriant et al. 2023^17^	Macrophage/slow-growing
THP-1 Normoxia	Evotec	Badé et al. 2021^18^	Macrophage/slow-growing
LCC	Literature search	Lakshminarayana et al. 2015,^19^ Sarathy et al. 2013^20^	Nutrient substitution/slow-growing
LORA MBC	TBDA	Cho et al. 2007^21^	Nutrient substitution/slow-growing
LORA MIC	TBDA	Cho et al. 2007^21^	Nutrient substitution/slow-growing
RAW264.7	Literature search	Lakshminarayana et al. 2015^19^	Macrophage/fast-growing
MBC	Literature search; Dartois Lab, HMH	Lakshminarayana et al. 2015,^19^ Sarathy et al. 2013,^20^ Koul 2008^22^	Nutrient-rich/fast-growing
MBC NR	Literature search	Xie et al. 2005,^23^ Upton et al. 2014,^10^ Sarathy et al. 2013^20^	Nutrient substitution/slow-growing
MIC	Literature search; Dartois Lab, HMH	Lakshminarayana et al. 2015,^19^ Cho et al. 2007,^21^ Silva et al. 2018,^24^ Kim 2021,^25^ Drusano 2018,^26^ Upton et al. 2014^10^	Nutrient-rich/fast-growing
MIC NR	Gold Lab, WCMC	Gold et al. 2015,^12^ Upton et al. 2014^10^	Nutrient substitution/slow-growing
NARA MBC	TBDA	Gold et al. 2015^12^	Nutrient-rich/fast-growing
Replicative Extracellular MIC	Evotec	Microplate Alamar Blue Assay in Zhang et al. 2023^27^	Nutrient substitution/fast-growing
NIAID Cholesterol	Boshoff lab, NIAID	Nuermberger et al. 2022^28^	Nutrient substitution/slow-growing
WCC	Literature search	Lakshminarayana et al. 2015,^19^ A. Koul 2008^22^	Nutrient substitution/slow-growing

NR, non-replicative; MIC, minimum inhibitory concentration; MBC, minimum bactericidal concentration; FBS, fetal bovine serum; BSA, bovine serum albumin; BCG, Bacille Calmette-Guérin; CARA, Charcoal Agar Resazurin Assay; NARA, normal agar resazurin assay; NIAID, National Institute of Allergy and Infectious Diseases; NR, non-replicative; LCC, Loebel cidal concentration; LORA, Low-Oxygen Recovery Assay; WCC, Wayne cidal concentration; TBDA, Tuberculosis Drug Accelerator; TU, Tufts University; HMH, Hackensack Meridian Health; WCMC, Weill Cornell Medical College.

### Feature selection of most informative *in vitro* assays

To reduce redundancy in the model, we carried out feature selection on all 31 assays, selecting those that had information on more than three drugs of interest, as well as those with a high number of pairwise correlations, which would be most representative of a large number of assays in the dataset (Figures 2B and 2C). A cutoff of >6 high pairwise correlations was selected, which gave us the upper 50% quantile of highly correlated assays. Table 2 lists each pairwise correlation for the assays with high numbers of pairwise correlations. Using pairwise correlation, we found 52 *in vitro* assay pairs with |R| >0.9, indicating a strong pairwise correlation. Four assays—caseum MBC, charcoal agar resazurin assay (CARA) MBC, low oxygen recovery assay (LORA) MIC, and intracellular potency assay in RAW264.7 macrophages—did not have high pairwise correlations with other assays and were therefore selected as informative features. Using network analysis, we found that a large cluster of highly correlated assays was formed with the remaining 25 assays. In this large cluster, eight assays with more than six pairwise correlations were chosen. The normal agar resazurin assay (NARA) MBC NR and the hypoxia-induced non-replication assay using the “Wayne cidal concentration” (WCC) as readout were also chosen, as they only had one high correlation with an assay in the large cluster (Figures 2B and 2C). In total, 14 *in vitro* assays were selected as features for further exploration.

**Table 2:** *In vitro* assays with six or more high correlations (|R| > 0.9)

Assays with most high correlations	Highly correlated assays	Total number of assays
Butyrate	Human Macrophage, Acidic, Tufts cholesterol, Tufts High cholesterol, Dormancy, Standard, NIAID cholesterol, 3 stress assay, Valerate	9
Tufts Cholesterol	Human Macrophage, Acidic, Butyrate, Tufts High cholesterol, Dormancy, Standard, NIAID cholesterol, 3 stress assay, Valerate	9
Dormancy	4 stress NR MIC, Human Macrophage, Acidic, Butyrate, Tufts cholesterol, Standard, NIAID cholesterol, 3 stress assay, Valerate	9
NIAID Cholesterol	4 stress NR MIC, Human Macrophage, Butyrate, Tufts cholesterol, Dormancy, Standard, 3 stress assay, Valerate	8
Valerate	Acidic, Butyrate, Tufts cholesterol, Tufts High cholesterol, Dormancy, J774, THP1 normoxia, NIAID cholesterol	8
Human Macrophage	Butyrate, Tufts cholesterol, Dormancy, Standard, NARA MBC, NIAID cholesterol, 3 stress assay	7
Acidic	Butyrate, Tufts cholesterol, Dormancy, J774, MBC, MIC, Valerate	7
3 Stress Assay	4 stress NR MIC, Human Macrophage, Butyrate, Tufts cholesterol, Dormancy, Standard, NIAID cholesterol	7
Standard	Human Macrophage, Butyrate, Tufts cholesterol, Dormancy, NIAID cholesterol, 3 stress assay	6

### Feature perturbation demonstrates that four or fewer *in vitro* assays can predict *in vivo* EC_50_ across mouse infection model types

Although data from three mouse models were available, we mainly discuss subacute mouse models in this text, as this was the mouse model previously used to validate our preclinical-to-clinical early bactericidal activity predictions.^9^ Acute and chronic mouse model results are described in Figures S1–S6.

Univariate analysis was first carried out to understand the individual relationships between *in vitro* EC_50_ and *in vivo* EC_50_ values (Figure S1). Likely due to the small size of the dataset, most univariate relationships were not statistically significant, leading us to perform feature perturbation instead.

**Figure 3:**
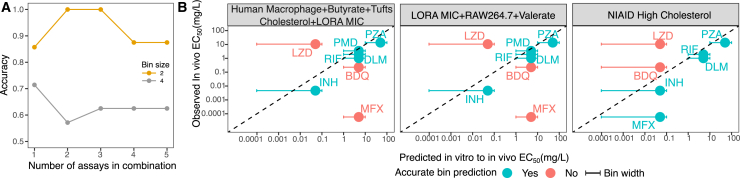
Feature perturbation identifies the best-performing combination of *in vitro* assays (A) Feature perturbation was carried out to find the minimum number of features that could reliably classify *in vivo* mouse EC_50_ from *in vitro* potencies. All possible combinations of 1–5 *in vitro* assays were carried out. (B) Test result of leave-one-out cross-validation performance in the training set with best-performing *in vitro* assay combinations. The lowest bin of <0.1 mg/L has an extended bin width to indicate that any drug with predicted EC_50_ < 0.1 mg/L will fall into that bin. BDQ, bedaquiline (BDQ), delamanid (DLM), ethambutol (EMB), isoniazid (INH), linezolid (LZD), moxifloxacin (MXF), pretomanid (PMD), pyrazinamide (PZA), rifampicin (RIF), and rifapentine (RPT).

Multivariate regression was unable to give good predictions using all 14 selected features due to overfitting of the training dataset. While the training dataset always gave an R^2^ of 0.99, the test drug EC_50_ was consistently predicted to be 100-fold or higher than its actual value. We therefore tried multinomial regression by classifying the *in vivo* EC_50_ values into four bins (very low, <0.1; low, 0.1–1; mid, 1–10; high, >10 mg/L). Although the test prediction improved slightly, it was still insufficient to get a reliable classification of the *in vivo* EC_50_. However, as we saw low-to-moderate correlations with *in vivo* mouse EC_50_ (Figure S1), there may have been multiple interacting features within the dataset, leading us to attempt feature perturbation (by creating all possible 1–5 combinations of *in vitro* assays based on the 14 assays from feature selection) to find the minimum number of features required to make a reliable prediction across two bins, low and high EC_50_. The model had a drop in accuracy from 100% to 62.5% when we further split EC_50_ predictions into all four bins, except for the NIAID cholesterol assay, which maintained its accuracy of 70% (Figure 3A). This allowed us to conclude that four or fewer assays were sufficient for the prediction of *in vivo* EC_50_.

We then compared the best-performing assay combinations against one another (Figure 3B). A four-assay combination of Human macrophage, Butyrate, Tufts cholesterol, and LORA MIC (62.5% accuracy), a three-assay combination of LORA MIC, RAW264.7, and valerate (62.5% accuracy), and single-assay NIAID high cholesterol (70% accuracy) were the top-performing assays. Across all the assays, the most difficult drug potencies to predict were for BDQ and LZD. LZD had a very different set of *in vitro* assays compared to other drugs in the same bin (DLM, PMD, and RIF), seen in Figure 2A. Here, LZD was on the extreme right of the clustered heatmap, whereas DLM, PMD, and RIF were clustered much more closely together, which could explain the difference in prediction. MFX, on the other hand, had an abnormally low *in vivo* EC_50_ of 0.0000586 mg/L (<0.1 bin), making it extremely difficult to predict compared to other drugs, as no other drugs had similar information. For BDQ, having an *in vivo* EC_50_ of 0.228 mg/L made it the only observed drug in the 0.1–1 bin. This led to all models having insufficient information for BDQ prediction in this analysis. In the next section’s external validation, however, we were able to predict new drugs in this 0.1–1 potency bin because the training set for that analysis included all eight drugs, including BDQ. Thus, we provide evidence that our top three models are reliable in making predictions for *in vivo* EC_50_. Our four-way *in vitro* assay combination of human macrophage, butyrate, BA cholesterol, and LORA MIC was preferentially chosen over top-performing single-assay combination NIAID high cholesterol based on the biological understanding of tuberculosis as a heterogeneous disease, making it crucial to incorporate multiple assays. Similar analyses for acute and chronic infection models are reported in Figure S3.

### Selected *in vitro* assay combinations can reasonably predict *in vivo* EC_50_ of new drugs

**Figure 4:**
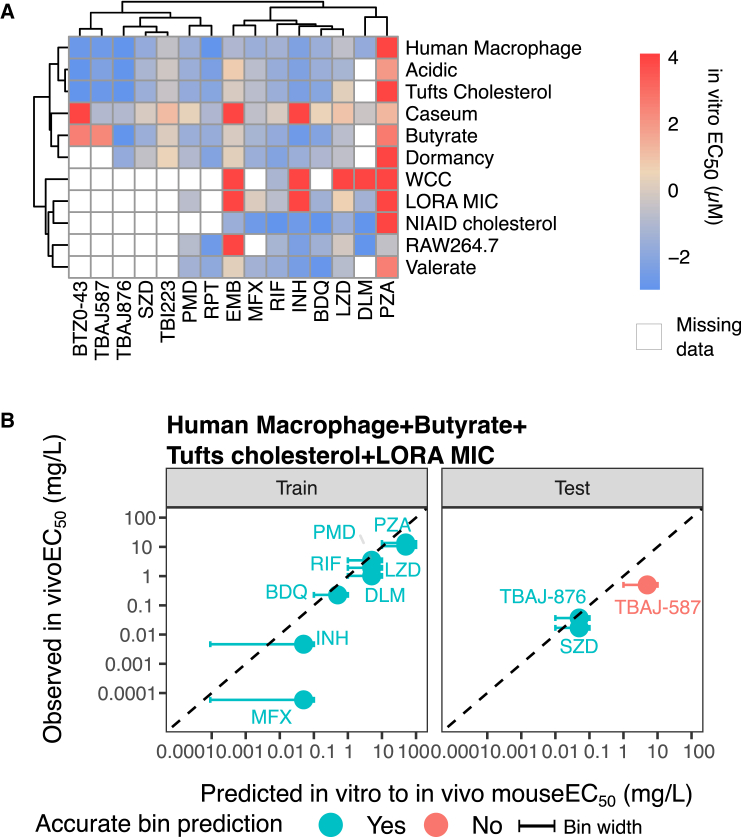
External validation of the model with TB drug candidates (A) Data availability of *in vitro* assays with five new TB drugs. (B) Model performance across different *in vitro* assay combinations shows that some assay combinations are more generalizable to new drugs. Error bars represent the bin width of the predicted bin, and points align with the observed *in vivo* EC_50_ on the y axis and middle of the predicted bin on the x axis. The lowest bin of <0.1 mg/L has an extended bin width to indicate that any drug with predicted EC_50_ < 0.1 mg/L will fall into that bin. The training set consisted of the 10 drugs used to train the models, whereas the testing set consisted of new drugs used to validate the models. BDQ, bedaquiline, DLM, delamanid, EMB, ethambutol, INH, isoniazid, LZD, linezolid, MXF, moxifloxacin, PMD, pretomanid, PZA, pyrazinamide, RIF, rifampicin, RPT, rifapentine, and SZD, sutezolid.

As an external validation, we compiled a list of top-performing assays and reached out to collaborators to test new compounds with these assays (Figure 4A). The new compounds were sutezolid (SZD), TBAJ-587, and TBAJ-876 for the subacute mouse model, all of which are drugs currently being developed for the treatment of TB. Acute and chronic mouse models also had EC_50_ predictions for SZD, TBI223, and BTZ-043 and are reported in Figure S4. The top-performing acute model was able to predict TBI223, but not SZD, correctly, whereas the chronic model was able to predict BTZ-043 accurately. As all three top-performing combinations performed similarly, the four-way *in vitro* assay combination of foamy and hypoxic human macrophages (human macrophage), changing the main carbon source to butyrate (butyrate), changing the main carbon source to cholesterol (Tufts cholesterol), and LORA MIC was preferentially chosen over other top-performing assay combinations based on data availability for the new drugs. Using the model trained on all eight TB drugs with subacute infection models, we inputted newly generated *in vitro* data from three new drugs into their respective trained mouse infection models. Out of three new drugs used for validation, SZD, TBAJ-587, and TBAJ-876, only TBAJ-587 was predicted incorrectly (predicted in the 1–10 bin, whereas observed EC_50_ was in the 0.1–1 bin) (Figure 4B). The results for acute and chronic models are available in Figure S4, with similar results.

### Binned EC_50_ values integrated with mouse PK and the bacterial dynamics model gave good predictions of mouse CFU profiles

**Figure 5:**
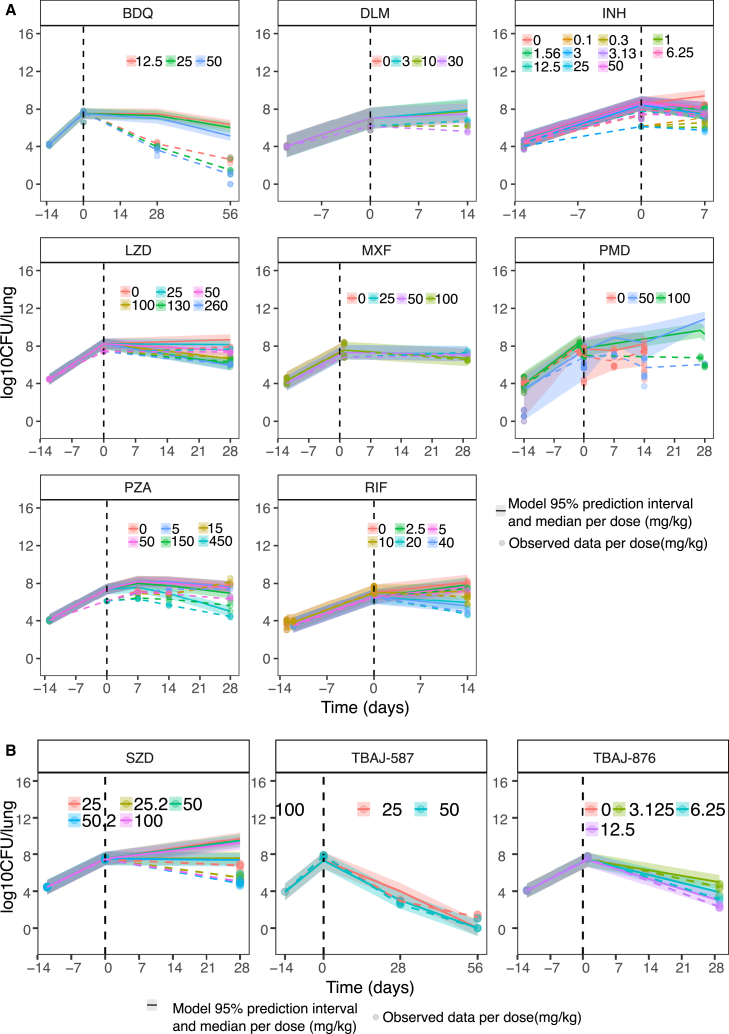
Simulations versus observed mouse data using predicted mouse EC_50_ Five hundred simulations per drug per mouse infection model were run. Ribbons represent the 95% prediction interval and solid lines the median model prediction. Dotted lines are the median of observed values. Observed data are represented as points. (A) Simulations with the initial eight drugs used for feature selection and model development. (B) Simulations with three new drugs used as external validation demonstrate the extent to which our model was generalizable. The *in vitro* to *in vivo* EC_50_ prediction models selected for this simulation of mouse CFU are as listed in Table S1. Model performance across all mouse infection models is in Figure S5. BDQ, bedaquiline, DLM, delamanid, EMB, ethambutol, INH, isoniazid, LZD, linezolid, MXF, moxifloxacin, PMD, pretomanid, PZA, pyrazinamide, RIF, rifampicin, RPT, rifapentine, and SZD, sutezolid.

After predicting the *in vivo* EC_50_ from the best-performing combination of *in vitro* assays, we took the median of each bin containing the predicted *in vivo* EC_50_, along with the median E_max_ per mouse infection model as the exposure-response relationship in mice, in our integrated PK-PD model with bacterial dynamics. This allowed us to predict the colony-forming unit (CFU) drop in mice over time, over a range of drug doses for all eight training drugs in the subacute mouse model (Figure 5A). The 95% prediction intervals from the models overlapped with observed mouse CFU data, indicating that the predictions were accurate. The only exception was BDQ, whose efficacy was overpredicted in mice. Similarly, we were able to capture the mouse profiles for new drug candidates SZD, TBAJ-587, and TBAJ-876 as validation of the model (Figure 5B).

### Predicted *in vivo* exposure-response informed the prediction of clinical EBA

**Figure 6:**
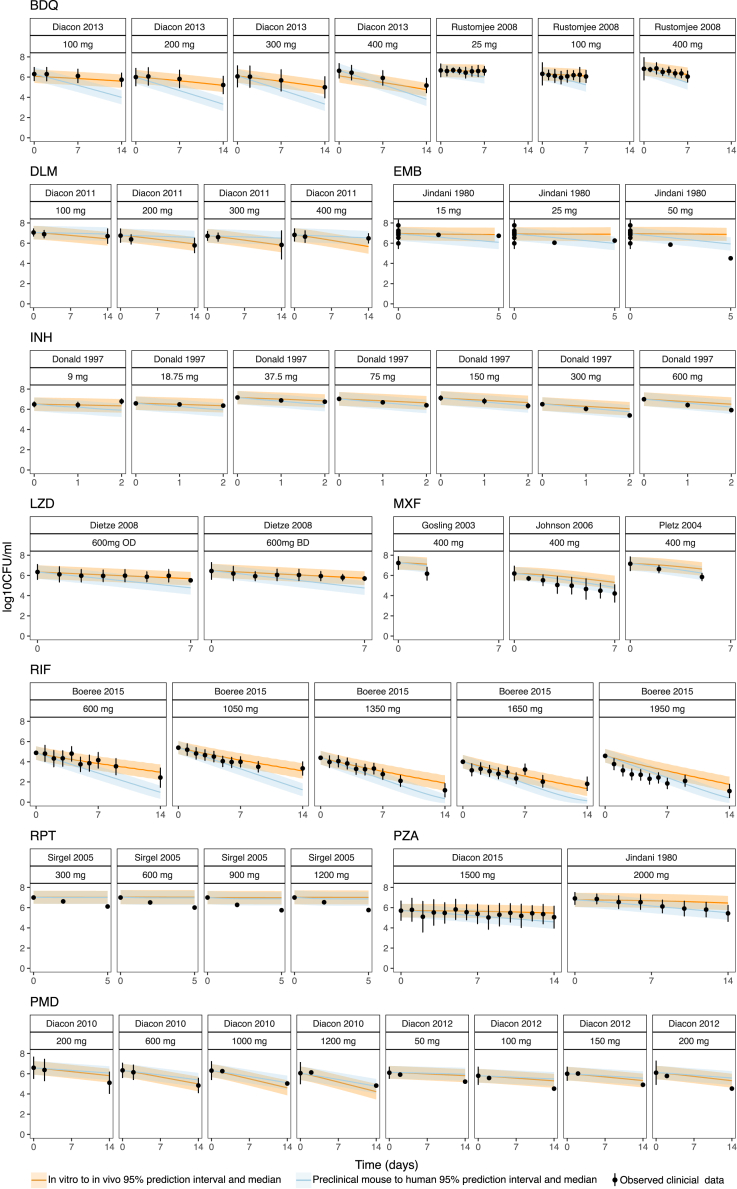
Prediction of early bactericidal activity outcomes in clinical populations Simulations using clinical PK with the predicted exposure-response relationship show good fits with observed clinical trial data for the 10 training set drugs. Subacute model predictions were used for all models except for EMB, which used an acute model and RPT, which used a chronic model. BDQ, bedaquiline, DLM, delamanid, EMB, ethambutol, INH, isoniazid, LZD, linezolid, MXF, moxifloxacin, PMD, pretomanid, PZA, pyrazinamide, RIF, rifampicin, RPT, rifapentine. Observations are represented as mean +/− standard deviation, and simulations are represented as mean with 95% confidence intervals.

Similarly, by combining clinical drug exposure with predicted exposure-response, we were able to get EBA predictions for all 10 drugs over 14 clinical trials (Figure 6). Our results were largely similar to preclinical mouse-to-human predictions, with most predictions overlapping well with the observed clinical data.^9^ Only BDQ had an overpredicted clinical EBA due to it being much more effective in a mouse model compared to clinical observations. However, our predicted *in vivo* exposure-response from *in vitro* data was able to capture the clinical observations more accurately, with higher overlap of the predictions and observations. This demonstrates that exposure-response relationships are translatable between systems.

## Discussion

We have systematically evaluated >30 *in vitro* assays used in TB drug development for their potential to predict *in vivo* outcome in mice and humans. Using *in vivo* EC_50_ as the primary outcome to identify which *in vitro* assays could predict mouse efficacy and best inform preclinical dosing, we were able to identify a set of four *in vitro* assays that could capture the *in vivo* potency well. This was then externally validated using three new drugs with good accuracy. When the predicted *in vivo* EC_50_ values were combined with our integrated bacterial dynamics PK-PD model, we were also able to capture the decrease in mouse CFU over time. This provides us with a framework for translating exposure-response relationships between different model systems, from *in vitro* to preclinical to clinical observations, which can guide dosing recommendations and prioritize drug candidates most likely to succeed in clinical trials.

*In vitro* assays are the least resource-intensive platform for gauging a compound’s potential in the discovery and development pipeline.^29^ However, *in vitro* drug potency should be coupled with drug exposure to fully capture the drivers of a drug’s *in vivo* efficacy. Thus, to translate these findings into interpretable biological outcomes, we combined predicted exposure-response relationships with either mouse or clinical PK data and identified a four-assay *in vitro* signature predictive of mouse bacterial burden upon treatment and short-term (Phase 2a) clinical outcomes. This is useful in prioritizing candidates for resource-intensive animal studies and suggesting reasonable first-in-human doses for clinical testing, provided they are safe.

We compiled data from a large panel of assays with differing complexities, from axenic culture in different types of culture media to macrophage infections and an *ex vivo* assay in rabbit caseum. Given that TB is a highly heterogeneous and dynamic disease over the course of infection,^30^^,^^31^^,^^32^ it is no surprise that multiple assays were required for the prediction of *in vivo* drug efficacy, especially with a mouse subacute model, where the adapted immune response has mounted, but bacteria are still actively replicating.^33^ Up to four *in vitro* assays describing key ecological niches were thus required.

Due to high correlation between *in vitro* assays, only a subset was required to describe the various pathophysiological states of Mtb. Both acute and subacute mouse infection models required more than one assay to have reliable prediction of *in vivo* EC_50_, suggesting that different assays describing different biological niches were required for *in vivo* EC_50_ prediction. Interestingly, the chronic mouse infection model was well described by the caseum assay alone, despite BALB/c mice not forming necrotic lesions.^34^^,^^35^ It is possible that after a long inoculation, the mouse lesions are still rich with foamy macrophages, whose lipid droplets are very similar in composition to caseum, found in the necrotic lesion core.^36^ This is consistent with *ex vivo* caseum as a single assay that reflects drug efficacy *in vivo*.^14^^,^^36^

We found that many *in vitro* assays where only the culture medium differed were highly correlated. However, none of these assays showed good correlation with the *ex vivo* caseum assay,^14^^,^^36^ which is currently understood as one of the most reflective of dormant Mtb bacilli found in hard-to-treat cavitary TB. This suggests that a single change in media formulation alone may not be sufficient to induce the same dormant state as seen within a necrotic TB lesion. Interestingly, the five macrophage assays were not strongly correlated to one another nor to the acidic *in vitro* assay meant to mimic the condition of Mtb surviving in the macrophage lysozyme, despite all macrophage assays expected to engulf Mtb into an acidic phagolysosome.^37^ This highlights that macrophage response in Mtb infection can be heterogeneous and/or differentially recapitulated *in vitro*, which has also been reported in other studies.^38^ We thus found that a multi-assay signature, rather than a single assay, could capture this physiological heterogeneity and predict *in vivo* EC_50_.

It was also interesting to note that despite both NIAID high cholesterol^28^ and Tufts high cholesterol^16^ having similar cholesterol concentrations (0.259 mM and 0.2 mM, respectively), the two assays had differences in media formulation and handling of the microbes after introduction to the culture prior to treatment initiation and reported different assay outcomes. This highlights that other biological factors might be at play in influencing Mtb and warrant further study.

To further test the utility of an *in vitro* to clinical EBA prediction, we similarly simulated clinical EBA predictions using the predicted *in vivo* EC_50_ alongside a clinical PK model and observed similarly good predictions to our translational mouse model predictions. Species-specific differences between mouse and human can exist, such as the case with BDQ, highlighting the potential utility of *in vitro* assays to overcome this limitation with those more tailored to human physiology.

### Limitations of the study

Our initial dataset was limited in that we only had 8 drugs against a total of 14 features. We thus employed leave-one-out cross-validation, instead of the traditional 80:20 training-to-testing ratio for model validation.^39^ Trying to predict *in vivo* EC_50_ as a continuous variable using multiple linear regression led to substantial model overfitting in the training set and poor predictions on the left-out drug. Binning the *in vivo* EC_50_ into ranges rather than discrete values provided a good estimate of *in vivo* EC_50_ and allowed for reliable predictions to be made on the left-out testing drug. Other machine learning methods such as random forest were also tried with poor accuracy and robustness, leaving us to use simple multimodal regression. The median of the bin served as a sufficient estimate for mouse CFU prediction, with the prediction of mouse bacterial burden with drug treatment overlapping well with observed experimental data.

A few drugs did not have good predictions in mice due to their *in vivo* E_max_ deviating greatly from the median E_max_ of all other drugs. Due to multiple assays in our database reporting only EC_50_ and not E_max_, we were unable to do a similar prediction for E_max_, which is a limitation of this study. However, the sensitivity analysis showed that simulated mouse CFUs were mostly similar, whether median E_max_ or actual model-derived E_max_ was used (Figure S6).

Our dataset was biased toward drugs that had already been approved or are in clinical development for TB. This was a limitation due to the number of new compounds we were able to get data for. Access to a broader range of agents to validate the pipeline, including discovery compounds that failed preclinical development for lack of efficacy, would be useful to test the robustness of the model and its utility for decision-making and is being planned for future study. This study was limited to single drugs and short-term EBA. Due to the lack of information about the fraction unbound of the drugs in each assay, we were unable to correct for fraction unbound or dynamic free fraction, which may help to improve the predictions. Further work is ongoing to predict the long-term efficacy of drug combinations and investigate the role of the fraction unbound and dynamic free fraction in prioritized assays.

Furthermore, although we could have generated efficacy predictions for single drugs, we wanted to ensure that the most predictive assays across drug classes and mechanisms of action should be prioritized as most informative assays. This study has shown that such *in vitro* to *in vivo* translations are promising, and further efforts to expand this database and model validation would be pertinent as novel compounds are developed.

## Resource availability

### Lead contact

Further information and requests for resources and reagents should be directed to, and will be fulfilled by, the lead contact Rada Savic (Rada.savic@ucsf.edu).

### Materials availability

This study did not generate new unique reagents.

### Data and code availability

• Data: the *in vitro* data used in this study are not made available due to confidentiality reasons but is available upon reasonable request from the authors with the approval of the individual asset holders.
• Code: all equations used to build models are listed in the supplementary section of this paper. The pipeline to select clinically relevant assays from mouse EC50 is located under https://github.com/JaniceGoh93/MTB_IVIVC. Any additional information required to reanalyze the data reported in this paper is available from the lead contact upon request.

## Acknowledgments

We wish to thank Carl Nathan, Benjamin Gold, and Helena Boshoff for sharing raw potency data. We would also like to thank Jacqueline Ernest, Belén Perez Solans, Annamarie Bustion, and Linda Chaba for their feedback and discussions on this manuscript.

This work was funded by award INV-002483 from the 10.13039/100000865Bill and Melinda Gates Foundation to V.D. and R.S., 10.13039/100000002NIH/NIAID UM1 AI179699 from the Preclinical Design and Clinical Translation of TB Regimens (PReDiCTR) Consortium, and INV-027276 from the Bill and Melinda Gates Foundation to B.B.A. The content is solely the responsibility of the authors and does not necessarily represent the official views of the National Institutes of Health.

## Author contributions

Conceptualization, J.J.N.G. and Q.W.W.; methodology and software, J.J.N.G.; formal analysis, J.J.N.G., A.P., B.N., R.C.W., N.S., and Q.W.W.; investigation and resources, J.J.N.G., N.V., T.M.W., V.D., J.S., B.B.A., and E.L.N.; data curation, J.J.N.G. and C.R.; writing—original draft, J.J.N.G. and V.D.; writing—review and editing, J.J.N.G., A.P., R.C.W., and V.D.; visualization, J.J.N.G.; supervision, R.M.S.; project administration, R.M.S.; funding acquisition, R.M.S., V.D., and B.B.A.

## Declaration of interests

The authors declare no conflicts of interest.

## STAR★Methods

### Key resources table

**Table:** 

REAGENT or RESOURCE	SOURCE	IDENTIFIER
**Software and algorithms**		

NONMEM 7.5.1	ICON Plc.	https://www.iconplc.com/solutions/technologies/nonmem
R 4.1.3.	The Comprehensive R Archive Network	https://cran.r-project.org/
Tidyverse	posit	https://www.tidyverse.org/
Pheatmap	Raivo Kolde	https://cran.r-project.org/web/packages/pheatmap/pheatmap.pdf
Nnet	Brian Ripley	https://cran.r-project.org/web/packages/nnet/nnet.pdf
Cor	Guangbao Guo	https://doi.org/10.32614/CRAN.package.COR

**Deposited data**		

*In vitro* assays	Multiple labs	Details and references in Table 1
Mouse PK and PD data	Multiple labs	Details and references in Table S1

### Experimental model and study participant details

#### Compiling a rich dataset of *in vitro* data

An initial set of drugs was selected based on the criteria that they had a corresponding integrated bacterial dynamics PK-PD model built across 3 different mouse infection models, acute, subacute, and chronic, and a minimum of 5 reported *in vitro* assay results. Detailed description of PK-PD model methods are described in Methods S1. For the sake of brevity, we focused on the subacute infection model, which was previously used to predict clinical Phase 2a outcomes. The same analyses were also carried out in acute and chronic mouse infection models, with their results reported in the supplemental information.

*In vitro* assays and their reported drug potencies (i.e., EC_50_) were compiled both from published literature and from collaborators (Table 1). To ensure fair comparison across drug potencies, all drug concentrations were standardized to mg/L and different measure outcomes (e.g., EC_50_, EC_80_, EC_99_) were converted to EC_50_ using the assumption that the Hill coefficient in a log-logistic dose-response curve was 1. After normalizing all values and doses across assays and drugs, curve fittings using both models with and without an estimated Hill coefficient were tested as a nested F test. When the percent activity was not stated for MIC, we assumed it to be EC_90_.

#### Building a translational mouse-to-human PK-PD dataset and model repository

Our model repository was built on rich longitudinal data pooled from available databases, including observed or published mouse PK data and mouse PD data (lung CFU counts) and human PD data (sputum CFU counts), as well as simulated human PK data using models as previously published.^40^ Multiple dose levels were investigated in both mouse PK and efficacy (PD) studies and human PK and EBA (PD) studies. All drugs were administered orally in mouse and human PK and PD studies. Mouse plasma samples were collected after either single or multiple doses of treatment, while human plasma samples were collected only after multiple doses.

Depending on the size of the infectious dose and the duration of incubation before treatment started, mouse efficacy studies were grouped as acute infection (inoculum size no less than 3.5 log_10_CFU/mL and incubation period no more than 8 days), subacute infection (inoculum size no less than 3.5 log_10_CFU/mL and incubation period between 10 and 17 days), and chronic infection studies (inoculum size less than 3.5 log_10_CFU/mL and incubation period no less than 21 days). Mice were dosed 5 days per week and CFU counts were collected 3 days after the last dose for any given mouse. In human EBA studies, the treatment duration ranged between 1 and 14 days. Integrated PK-PD models with bacterial dynamics were applied to the mouse data to elucidate the *in vivo* exposure-response using NONMEM 7.5.1 and Perl-Speaks-NONMEM (PsN) 5.3.0. Detailed methods on model building can be found in Ernest et al.^9^

### Quantification and statistical analysis

#### Exploratory data analysis and determination of translatable assays

To first assess data availability and spread of EC_50_ values across different assays, initial exploratory analyses were done using tidyverse, pheatmap, drc, nnet, and cor R packages using R 4.1.3. Feature selection was carried out based on the following criteria (Figure 2): 1) the *in vitro* assay contained information for at least 3 drugs of interest, and 2) between highly correlated assays, the most representative assay with the most pairwise correlations was chosen.

While initial clustering and data exploration were done in μM, we decided to do the binning and predictions of EC_50_ in mg/L, as that is the concentration that is commonly reported for PK studies, and we wanted to be as consistent as possible with published models. Because the main driver of the drug’s effect is its exposure characterized as mg/L over time, rather than its potency directly, the predictions in mg/L are more translatable for directly adding into a PK-PD model without additional unit conversions.

*In vitro* assays were first evaluated individually using univariate linear regression. Models to predict *in vivo* mouse EC_50_ were built individually for each mouse infection type as the *in vivo* EC_50_ trends changed between infection models for the same drug. As the potency of inactive compounds was unknown, inactive compounds were excluded from the univariate analysis.

Feature selection was first carried out by performing pairwise correlations using Pearson’s correlation between all *in vitro* assays. Assays with no high pairwise correlations were selected as features, and from groups of assays with high pairwise correlations (R>0.9), one representative assay with the most correlations was chosen.

Feature perturbation to test which combination of *in vitro* assays was most informative using multinomial linear regression was then applied to the dataset to predict *in vivo* EC_50_ from *in vitro* EC_50_. All possible 1-5-way combinations of *in vitro* assays were generated for the evaluation of predictive *in vitro* assay combinations. As initial analyses found multiple linear regression to have large model overfitting, we applied multinomial regression instead, using bins of very low (<0.1 mg/L), low (0.1-1 mg/L), mid (1-10 mg/L), and high (>10 mg/L) concentration ranges to classify EC_50_ values. Due to the small size of the dataset, we applied leave-one-out cross-validation to evaluate model accuracy instead of K-fold validation. Accuracy was defined as the number of times the left-out drug was predicted in the correct bin for multinomial linear regression. For drugs with missing assay values, the assay values were imputed as the median potency of all other drugs within the same *in vitro* assay.^41^

#### Combining PK models from mice with *in vitro*-*in vivo* (IVIV)-predicted exposure-response

To find out how well IVIV-predicted exposure-response mapped to drug efficacy in mice, we substituted predicted EC_50_ values into previously validated mouse PK models. The E_max_ in each exposure-response curve was estimated as the median E_max_ of all 10 drugs for each mouse infection model. This was done as most *in vitro* assays reported only a single EC_50_ value rather than concentration-response curves. A sensitivity analysis comparing simulations done using either the actual PK-PD model’s E_max_ or median E_max_ values are detailed in Figure S6. Simulations of bacterial burden (CFU) in mice over time were done using both the raw EC_50_ values for selected *in vitro* assays as well as the median of the binned EC_50_ values as predicted by the multinomial regression algorithm. Typical bacterial dynamic parameters were used as detailed in Zhang et al.^33^ The PK-PD simulations were done using NONMEM 7.5.1. Variability was simulated using variance in CFU at day 1 post inoculation in mice for acute, subacute, and chronic mouse infection models respectively, before treatment. Model predictions were evaluated using visual predictive checks (VPCs), by overlaying the median and 95% prediction interval of the model over observed results from mouse studies. Models were considered appropriate when prediction intervals overlapped well with the observed data. Equations and a detailed methodology are described in supplementary methods.

#### Combining clinical PK models with IVIV-predicted exposure response

To simulate clinical EBA, the predicted *in vivo* exposure-response relationship was combined with previously validated population PK models from literature to build a PK-PD model that was then used for simulation.^9^ Again, our models were evaluated using VPCs against observed clinical EBA data from 14 different studies for all 10 drugs. The subacute model results were used for all drugs except for EMB, which used acute model results, and RPT, which used chronic model results, as these drugs did not have subacute mouse data. Details and references of the clinical EBA studies are in Table S2.

#### External model validation with a set of new TB drugs

To externally validate the model, we compiled a list of the best-performing combinations of *in vitro* assays from initial multinomial classification and reached out to the same labs that made these assays to test 5 new compounds. We then input the *in vitro* EC_50_ values into the multinomial classifier trained using the original model to predict mouse EC_50_ for these new compounds. Detailed information on the mouse PD data used for validation and the mouse PK models used in the prediction are listed in Table S1.
