# Supplementary material for: Predicting tuberculosis drug efficacy in preclinical and clinical models from in vitro data
Source: iScience. 2025 Jan 30;28(3):111932. doi: 10.1016/j.isci.2025.111932 (PMC11875147; doi:10.1016/j.isci.2025.111932)
Supplement: Document S1. Figures S1–S6, Tables S1 and S2, and Methods S1 [file mmc1.pdf]

iScience, Volume *28*

Supplemental information

Predicting tuberculosis drug efficacy

in preclinical and clinical models

from *in vitro* data

Janice J.N. Goh, Anu Patel, Bernard Ngara, Rob C. van Wijk, Natasha Strydom, Qianwen Wang, Nhi Van, Tracy M. Washington, Eric L. Nuermberger, Bree B. Aldridge, Christine Roubert, Jansy Sarathy, Véronique Dartois, and Rada M. Savic

Methods S1

Mouse PK and PK/PD Model Development (Figures 1, 5, 6)

Mouse PK models of nine TB drugs, including BDQ, DLM, INH, LZD, MFX, PMD, PZA, RIF, and RPT, were developed using plasma concentration data individually, among which partial data for DLM were digitized from a published study (3 and 30 mg/kg) (9). Either a one-compartment or two-compartment structural model with first-order absorption and linear or non-linear clearance was used to describe the mouse PK data for each drug **(Table S1)** **(*Eq.* S1-S7)**. Saturable bioavailability was incorporated for PMD and RIF PK models ***(Eq.* S7).**

First-order absorption model:

$$\frac{dA_1}{dt} = -K_a \times A_1 \qquad \boldsymbol{Eq. S1}$$

A_1: amount of drug in the gastrointestinal tract absorbed into the systemic circulation
K_a: first-order absorption rate of the drug
t: time after dose

One-compartment PK model:

$$\frac{dA_2}{dt} = K_a \times A_1 - K_e \times A_2 \qquad \boldsymbol{Eq. S2}$$

A_2: amount of drug in the central compartment
K_e: elimination rate of the drug from the central compartment

Two-compartment PK model:

$$\frac{dA_2}{dt} = K_a \times A_1 - K_e \times A_2 - \frac{Q}{V_1} \times A_2 + \frac{Q}{V_2} \times A_3 \qquad \boldsymbol{Eq. S3}$$

$$\frac{dA_3}{dt} = \frac{Q}{V_1} \times A_2 - \frac{Q}{V_2} \times A_3 \qquad \boldsymbol{Eq. S4}$$

A_3: amount of drug in the peripheral compartment
Q: intercompartmental clearance
V_1: volume of the central compartment
V_2: volume of the peripheral compartment

Linear clearance:

$$K_e = \frac{CL}{V_1} \qquad \boldsymbol{Eq. S5}$$

CL: clearance, defined as the volume of plasma completely cleared of drug per unit time

Non-linear clearance:

$$K_e = \frac{K_m \times CL_{int}}{\left(K_m + \frac{A_2}{V_1}\right) \times V_1} \qquad \boldsymbol{Eq. S6}$$

V_{max}: maximal clearance, defined as the maximal volume of plasma completely cleared of drug per unit time
K_m: concentration of drug that results in half of the maximal clearance
CL_{int}: ratio between V_{max} and K_m.

Saturable bioavailability:

$$F = 1 - \frac{F_{DIF} \times (Dose - Dose_{ref})}{Dose - Dose_{ref} + FD_{50}} \qquad \boldsymbol{Eq. S7}$$

F: bioavailability, defined as the extent of drug absorbed from oral dosing compartment into systemic compartment
F_{DIF}: maximum difference in bioavailability from 100% (bound between 0% and 100%)
$Dose_{ref}$: reference dose that has 100% bioavailability
FD_{50}: dose achieving half maximal reduction in bioavailability

Similar to our previously published translation of drug monotherapy PK-PD from mouse to human, we used the same bacterial dynamics model to account for mouse immune response **(*Eq.* S8)**. Drug effect (EFF) was defined as bacterial killing rate (day^{-1}) and as the exposure-response of the drug of interest using the predicted in vivo EC_{50} from in vitro assays, and the E_{max} as the median E_{max} of all training drugs available **(*Eq.* S9)**. The median for each infection model was 0.968 days^{-1} for acute, 0.594 days^{-1} for subacute, and 0.421 days^{-1} for chronic infection. All effect models were assumed to be direct effect models. The observed output, bacterial number (B), was defined as the remaining bacteria after accounting for the dynamic processes of bacterial growth (K_g), natural bacterial death (K_d), and drug-induced bacterial death (EFF) **(Eq. S10).** For the prediction of mouse CFU, mouse plasma concentration was used, while for clinical Phase 2a predictions, clinical plasma concentration was used.

$$\frac{dB}{dt} = K_g \times B \times \left(1 - \frac{K_B \times B^{\gamma_B}}{B_{50}^{\gamma_B} + B^{\gamma_B}}\right) \times \left(1 - \frac{K_T \times t^{\gamma_T}}{T_{50}{}^{\gamma_T} + t^{\gamma_T}}\right) - K_d \times B \qquad \boldsymbol{Eq. S8}$$

$$EFF = \frac{E_{max,infection\ median} \times C_P^{\gamma}}{EC_{50,IVIVC}^{\gamma} + C_P^{\gamma}} \qquad \boldsymbol{Eq. S9}$$

$$\frac{dB}{dt} = K_g \times B \times \left(1 - \frac{K_B \times B^{\gamma_B}}{B_{50}^{\gamma_B} + B^{\gamma_B}}\right) \times \left(1 - \frac{K_T \times t^{\gamma_T}}{T_{50}{}^{\gamma_T} + t^{\gamma_T}}\right) - K_d \times B - EFF \times B \qquad \boldsymbol{Eq. S10}$$

B: *bacterial number*
t: *incubation time since inoculation*
K_g: *bacterial natural growth rate*
K_d: *bacterial natural death rate*
K_B: *bacterial number-dependent maximal adaptive immune effect*
B_{50}: *bacterial number that results in half of* K_B
γ_B: *steepness of bacterial number-dependent immune effect relationship*
K_T: *incubation time-dependent maximal adaptive immune effect*
T_{50}: *bacterial number that results half of* K_T
γ_T: *steepness of time-dependent immune effect relationship*
EFF: *bacterial killing rate*
$E_{max,infection\ median}$: *median maximal level of drug effect across all training drugs within the same mouse infection model*
$EC_{50,IVIVC}$: *plasma drug concentration that results in half of the maximal drug combination effect as predicted from in vitro assays*
C_P: *plasma drug concentration*

Supplementary Figures

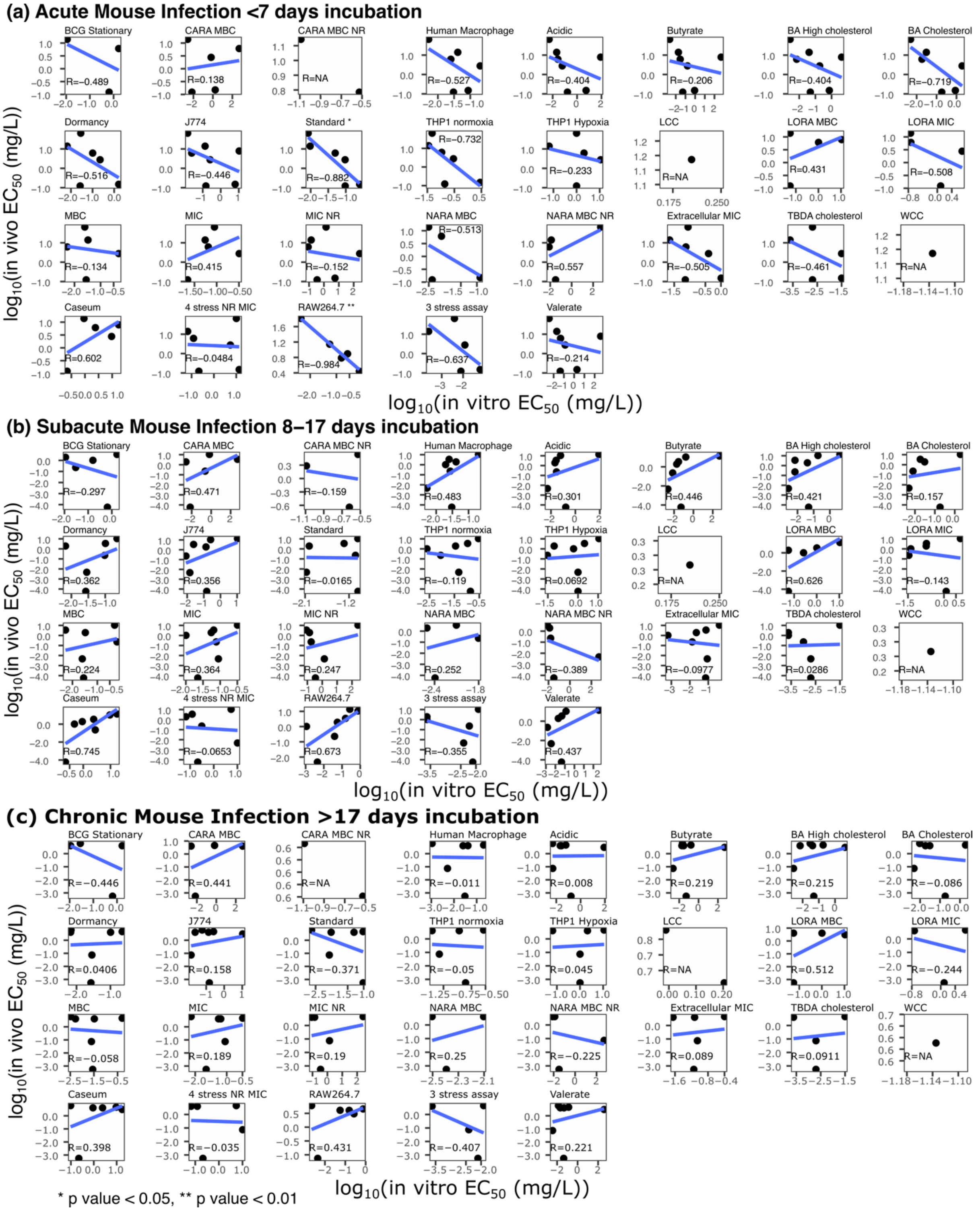

Figure S1. All 32 univariate correlations of in vitro versus in vivo EC_{50}. Inactive drugs were excluded from this analysis, leaving 29 plots. (a) In vitro EC_{50} against all in vivo EC_{50}s derived from acute mouse infection models. Acute mouse infection models had mice infected with H37Rv for 7 or less days prior to starting drug treatment. The drugs used in this analysis were EMB, INH, LZD, MFX, PMD, PZA and RIF. (b) In vitro EC_{50} against all in vivo EC_{50}s derived from subacute mouse infection models. Subacute mouse infection models had mice infected with H37Rv for more than 7 but less than 17 days prior to starting drug treatment. The drugs used in this analysis were BDQ, DLM, INH, LZD, MFX, PMD, PZA and RIF. (c) In vitro EC_{50} against all in vivo EC_{50}s derived from chronic mouse infection models. Chronic mouse infection models had mice infected with H37Rv for at least 21 days prior to starting drug treatment. The drugs used in this analysis were INH, LZD, MFX, PMD, PZA, RIF and RPT.

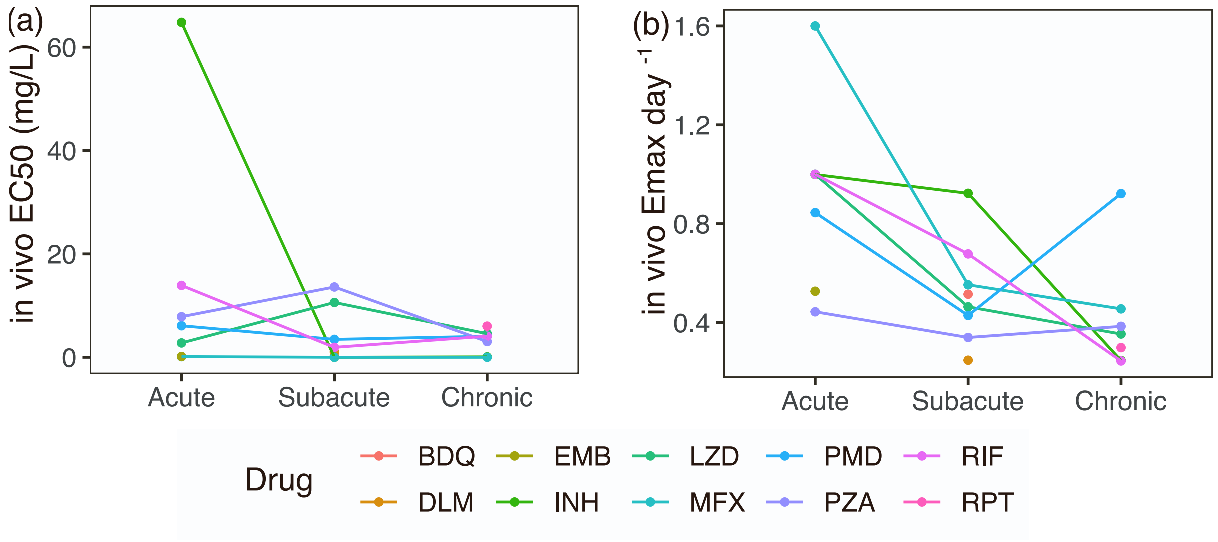

Figure S2. EC_{50} and E_{max} trends across mouse infection models. (a) In vivo EC_{50} does not have a trend across infection models. (b) In vivo E_{max} shows a trend toward lower E_{max} values with longer incubation time prior to treatment start.

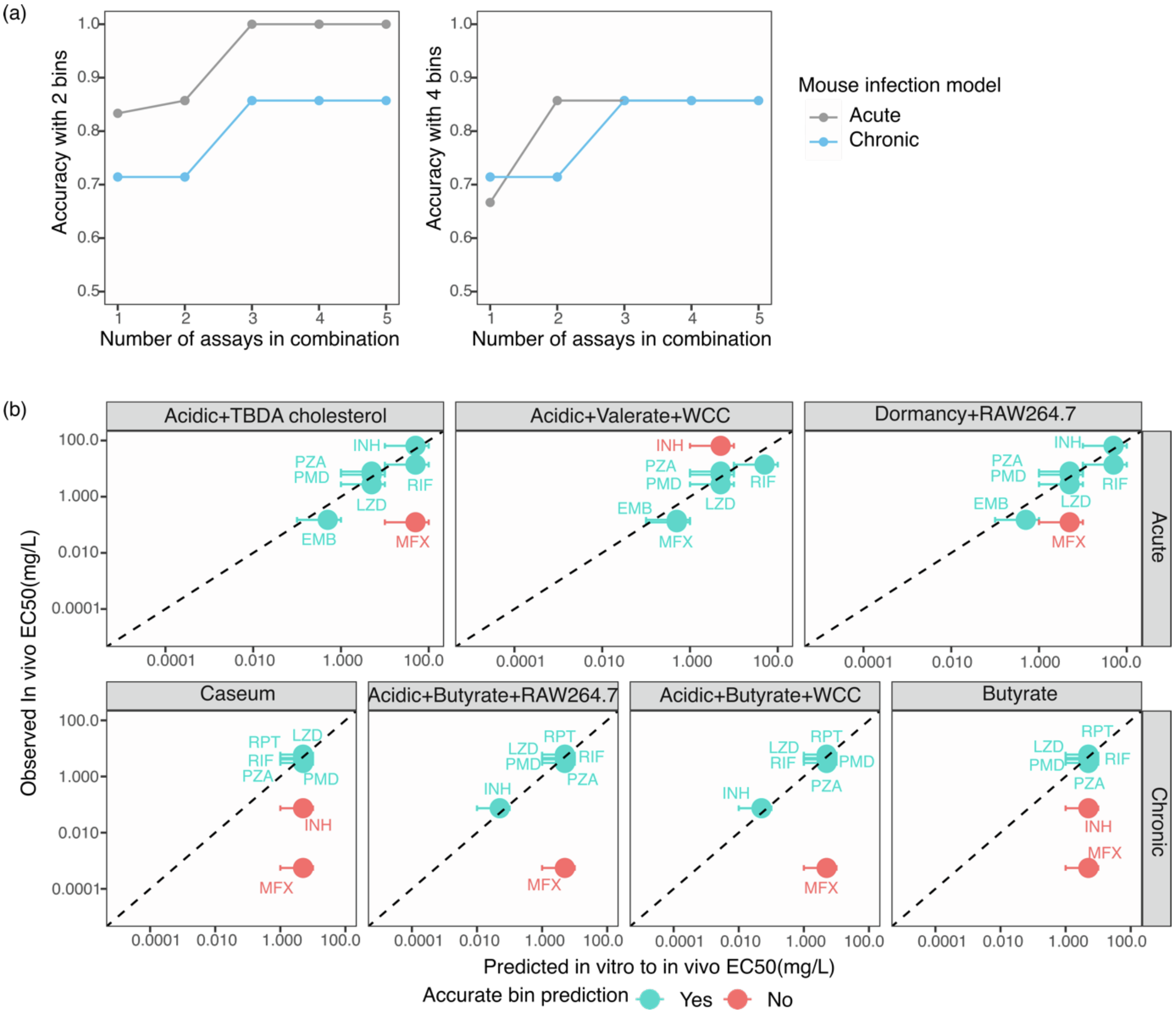

Figure S3. Test results of leave-one-out cross-validation performance in the training set with best performing in vitro assay combinations for acute and chronic mouse infection models.

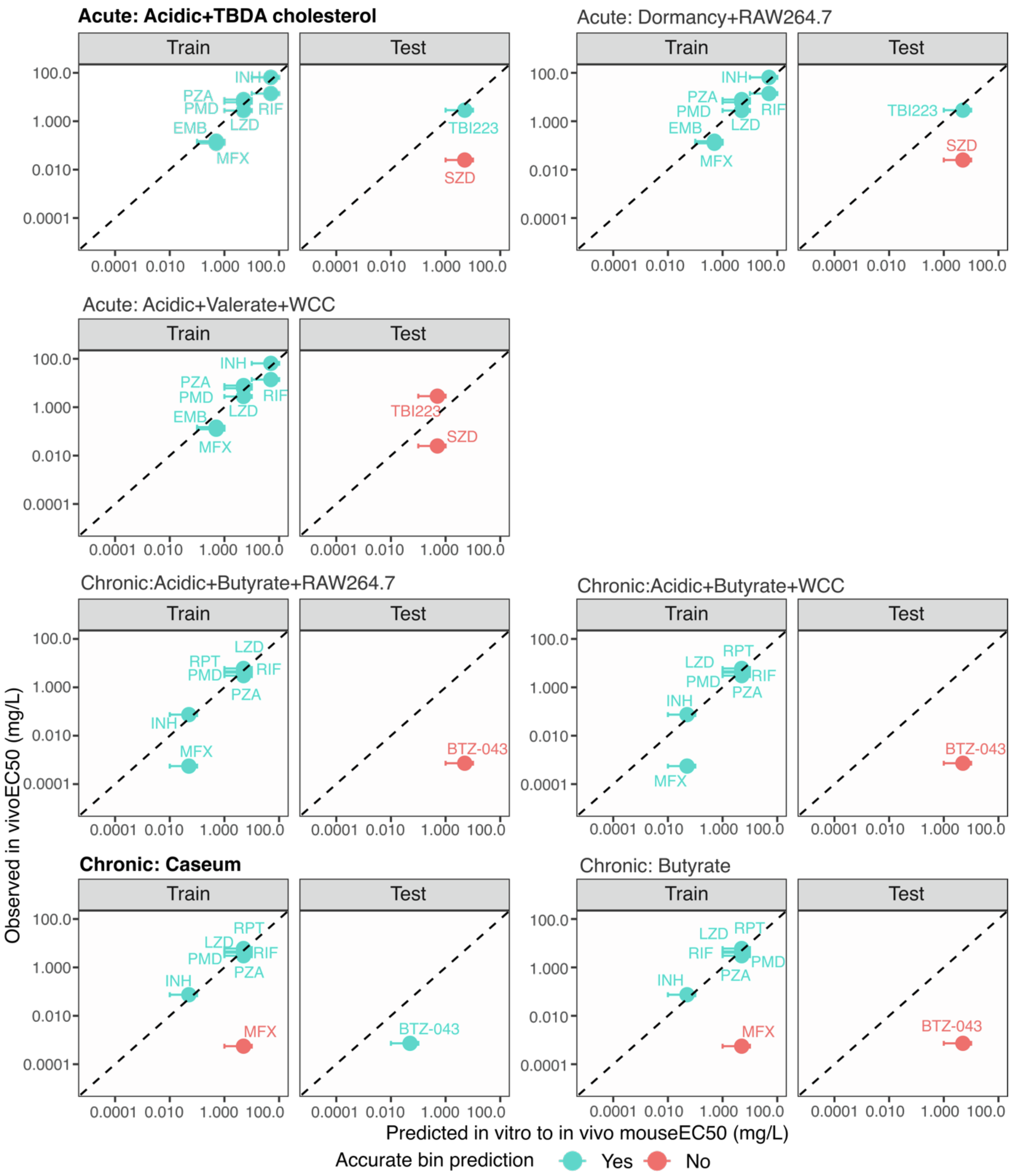

Figure S4. Model performance across different in vitro assay combinations shows some assay combinations are more generalizable to new drugs. Error bars represent the bin width of the predicted bin, and points align with the observed in vivo EC_{50} on the y-axis and middle of the predicted bin on the x-axis. The training set consisted of the 7 drugs used to train the models for both acute and chronic infection models, while the testing set contained new drugs used to validate the models. New drugs tested in acute models include TBI223 and SZD, while the new drug tested in chronic model was BTZ-043. Bolded combinations are the best performing assays for each model type.

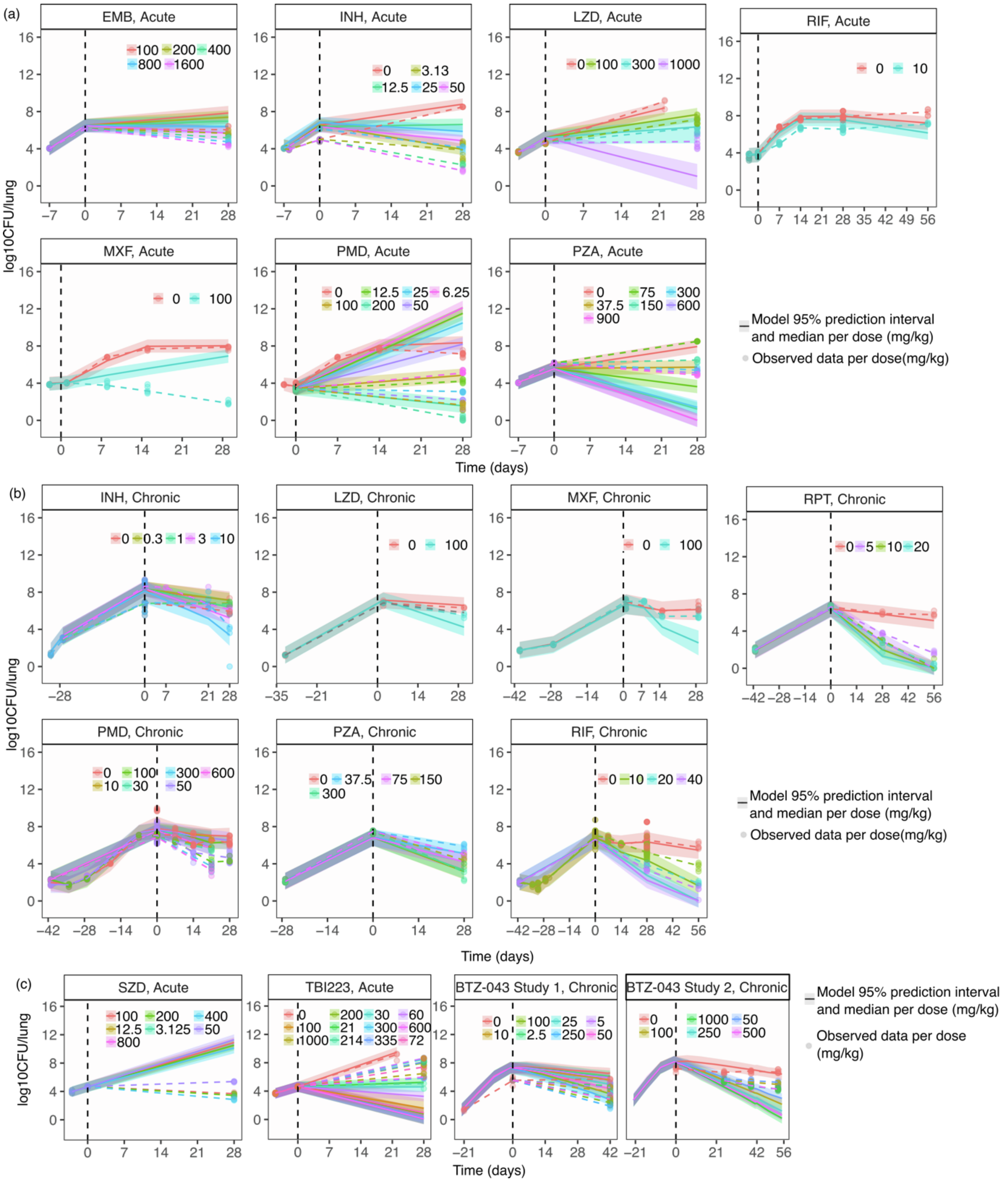

Figure S5. Simulations using predicted mouse EC_{50} overlap well with observed mouse data. 500 simulations per drug per mouse infection model were run. Ribbons represent the 95% prediction interval and solid lines the median model prediction. Dotted lines are the median of observed values. Observed data are represented as points. (a) Simulations with the initial 7 drugs used for feature selection and model development for acute mouse infection model. (b) Simulations with the initial 7 drugs used for feature selection and model development for chronic mouse infection model. (c) Simulations with 3 new drugs used as external validation demonstrate the extent to which our model was generalizable in acute and chronic mouse models. The in vitro to in vivo EC_{50} prediction models selected for this simulation of mouse CFU over time are as listed in **Table S1.**

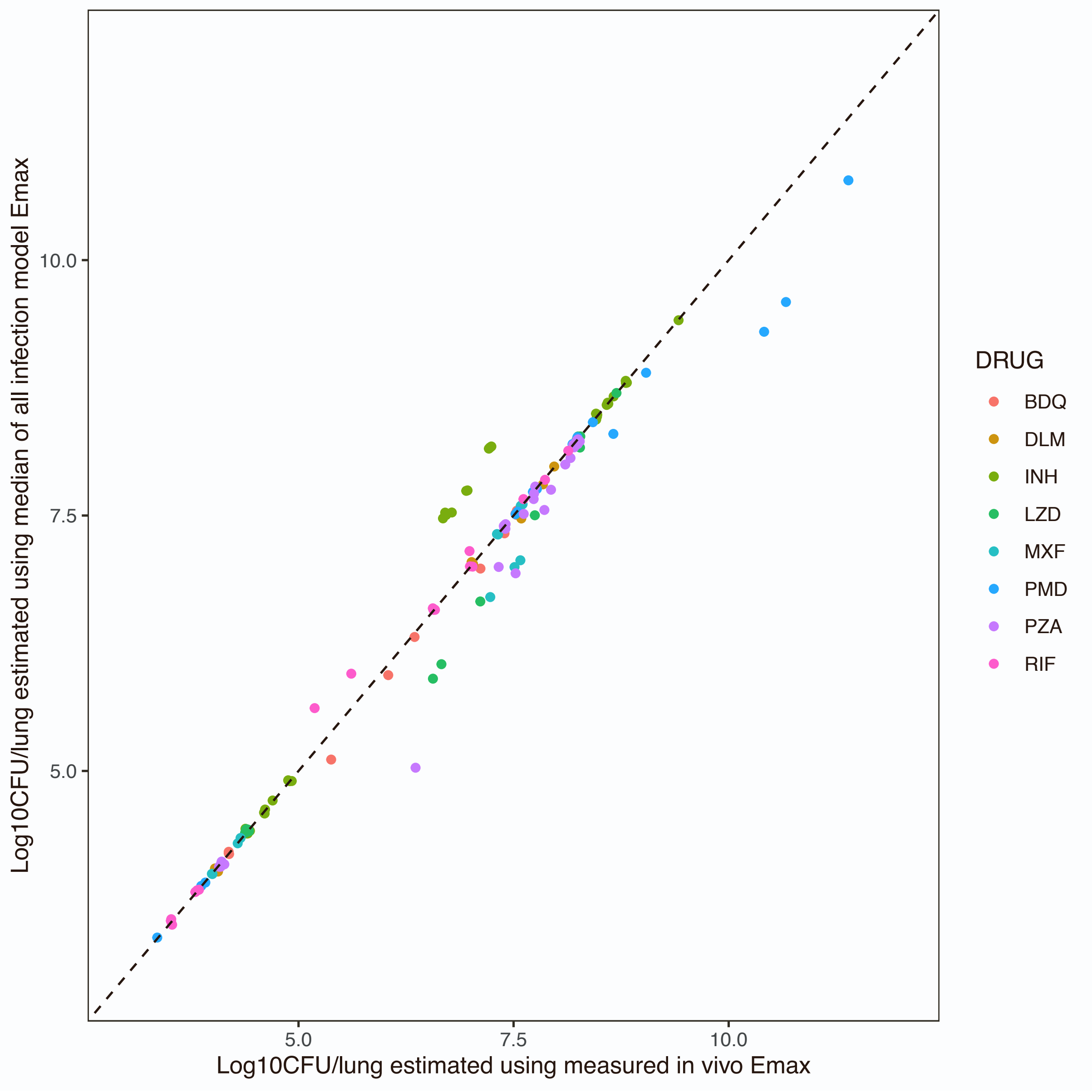

Figure S6. Comparison of simulated mouse CFU after treatment using predicted in vitro potency and either median E_{max} across infection models, or the actual model-derived in vivo E_{max}. Majority of the points fall along the line of unity, suggesting that a median E_{max} is sufficient to predict changes in mouse CFU.

Table S1. Mouse PK and PD data for new TB drug candidates for model building.

Drug	Source	PK dataset details	PD dataset details	Model exposure-response parameters	IVIVC-predicted exposure-response parameters
BTZ-043	University of Munich	86 observations<br>2.5, 5, 25, 50, 100, 250 mg/kg doses	**Chronic infection**<br>176 observations<br>0, 2.5, 5, 10, 25, 50, 100, 250, 500, 1000 mg/kg doses	Emax 0.188 day^{-1}<br>EC_{50} 0.000729 mg/L	Emax 0.421 day^{-1}<br>EC_{50} 0.05 mg/L<br>**Assay combination:**<br>Chronic model- caseum
TBAJ-876	John Hopkins University	231 observations<br>1, 2.5, 3, 3.125, 6.25, 10, 12.5, 25 mg/kg doses	**Subacute infection**<br>33 observations<br>0, 3.125, 6.25, 12.5 mg/kg doses	Emax 0.653 day^{-1}<br>EC_{50} 0.0368 mg/L	Emax 0.594 day^{-1}<br>EC_{50} 0.05 mg/L<br>**Assay combination:**<br>Subacute model- Human macrophage+Butyrate+BA Cholesterol+LORA MIC
TBAJ-587	John Hopkins University	333 observations<br>2.5, 6.25, 10, 12.5, 20, 25, 50, 100 mg/kg doses	**Subacute infection**<br>32 observations<br>25, 50 mg/kg doses	Emax 0.594 day^{-1}<br>FIX<br>EC_{50} 0.178 mg/L	Emax 0.594 day^{-1}<br>EC_{50} 0.5 mg/L<br>**Assay combination:**<br>Subacute model- Human macrophage+Butyrate+BA Cholesterol+LORA MIC
SZD	John Hopkins University	87 observations<br>50, 100 mg/kg doses	**Acute infection**<br>48 observations<br>50, 100, 200, 400 mg/kg<br><br>**Subacute infection**<br>75 observations<br>25, 25 BID, 50, 50 BID, 100 mg/kg	**Acute infection**<br>Emax 0.85 day^{-1}<br>EC_{50} 0.052 mg/L<br>Gamma 2.47<br>K_{delay} 0.27 day^{-1}<br><br>**Subacute infection**<br>Emax 0.71 day^{-1}<br>EC_{50} 0.025 mg/L<br>Gamma 2.27<br>K_{delay} 0.29 day^{-1}	**Acute infection**<br>Emax 0.968 day^{-1}<br>EC_{50} 0.05 mg/L<br>**Assay combination:**<br>Acute model- Acidic+TBDA cholesterol<br><br>**Subacute infection**<br>Emax 0.594 day^{-1}<br>EC_{50} 0.05 mg/L<br>**Assay combination:**<br>Subacute model- Human macrophage+Butyrate+BA Cholesterol+LORA MIC
TBI223	John Hopkins University	141 observations<br>3, 5, 100 mg/kg doses	**Acute infection**<br>261 observations<br>0, 300, 1000, 3000 mg/kg	Emax 0.444 day^{-1}<br>EC_{50} 2.86 mg/L<br>Gamma 0.623<br>K_{delay} 98.1 day^{-1}	Emax 0.968 day^{-1}<br>EC_{50} 5 mg/L<br>**Assay combination:**<br>Acute model- Acidic+TBDA cholesterol

Table S2. Human EBA studies

Drug	Doses	Baseline (log_{10} CFU/ml)	References
BDQ	100, 200, 300 and 400 mg (with 200, 400, 500, 700 mg loading dose on first day and 100, 300, 400, 500 mg on second day, respectively) 25, 100, 400 mg	6.302 (100 mg), 6.001 (200 mg), 6.071 (300 mg), 6.625 (400 mg) 6.66 (25 mg), 6.32 (100 mg), 6.82 (400 mg)	Diacon, A. H. et al. Antimicrob. Agents Chemother. 57, 2199–2203 (2013), Rustomjee, R. et al. Antimicrob. Agents Chemother. 52, 2831–2835 (2008).
DLM	100, 200, 300 and 400 mg	7.06 (100 mg), 6.75 (200 mg), 6.72 (300 mg), 6.82 (400 mg)	Diacon, A. H. et al. Int. J. Tuberc. Lung Dis. 15, 949–954 (2011).
EMB	15, 25, and 50 mg/kg	6.92	Jindani, A., Aber, V. R., Edwards, E. A. & Mitchison, D. A. Am. Rev. Respir. Dis. 121, 939–949 (1980).
INH	9, 18.75, 37.5, 75, 150, 300 and 600 mg	6.491 (9 mg), 6.585 (18.75 mg), 7.169 (37.5 mg), 7.031 (75 mg), 7.115 (150 mg), 6.504 (300 mg), 6.995 (600 mg)	Donald, P. R. et al. Am. J. Respir. Crit. Care Med. 156, 895–900 (1997).
LZD	600 mg QD, 600 mg BD	6.34 (600 mg QD), 6.44 (600 mg BD)	Dietze, R. et al. Am. J. Respir. Crit. Care Med. 178, 1180–1185 (2008)
MXF	400 mg	6.19 (400 mg Johnson), 7.15 (400 mg Pletz), 7.23 (400 mg Gosling)	Gosling, R. D. et al. Am. J. Respir. Crit. Care Med. 168, 1342–1345 (2003), Johnson, J. L. et al. Int. J. Tuberc. Lung Dis. 10, 605– 612 (2006), Pletz, M. W. R. et al. Antimicrob. Agents Chemother. 48, 780–782 (2004).
PMD	50, 100, 150, 200, 600, 1000, 1200 mg	6.1 (50 mg), 5.8 (100 mg), 6 (150 mg), 6.1 (200 mg Diacon 2012), 6.592 (200 mg Diacon 2010), 6.335 (600 mg), 6.309 (1000 mg), 6.057 (1200 mg)	Diacon, A. H. et al. Antimicrob. Agents Chemother. 54, 3402–3407 (2010), Diacon, A. H. et al. Antimicrob. Agents Chemother. 56, 3027–3031 (2012).
PZA	1500, 2000 mg	5.56 (1500mg), 6.910 (2000mg)	Jindani, A., Aber, V. R., Edwards, E. A. & Mitchison, D. A. Am. Rev. Respir. Dis. 121, 939–949 (1980), Diacon, A. H. et al. Am. J. Respir. Crit. Care Med. 191, 943– 953 (2015)
RIF	10, 20, 25, 30 and 35 mg/kg	4.88 (10 mg/kg), 4.00 (20 mg/kg), 5.39 (25 mg/kg), 4.58 (30 mg/kg), 4.39 (35 mg/kg)	de Steenwinkel, J. E. M. et al. Am. J. Respir. Crit. Care Med. 187, 1127–1134 (2013).
RPT	300, 600, 900, 1200 mg	N/A	Sirgel, F. A. et al. Am. J. Respir. Crit. Care Med. 172, 128–135 (2005)
